# *In Vitro* Expansion of CAG, CAA, and Mixed CAG/CAA Repeats

**DOI:** 10.3390/ijms160818741

**Published:** 2015-08-11

**Authors:** Grzegorz Figura, Edyta Koscianska, Wlodzimierz J. Krzyzosiak

**Affiliations:** Department of Molecular Biomedicine, Institute of Bioorganic Chemistry, Polish Academy of Sciences, Noskowskiego 12/14 Str., 61-704 Poznan, Poland; E-Mails: gfigura@man.poznan.pl (G.F.); edytak@ibch.poznan.pl (E.K.)

**Keywords:** SLIP, trinucleotide repeats, *in vitro* expansion, repeats *in vitro* cloning, polyglutamine diseases, plasmid constructs

## Abstract

Polyglutamine diseases, including Huntington’s disease and a number of spinocerebellar ataxias, are caused by expanded CAG repeats that are located in translated sequences of individual, functionally-unrelated genes. Only mutant proteins containing polyglutamine expansions have long been thought to be pathogenic, but recent evidence has implicated mutant transcripts containing long CAG repeats in pathogenic processes. The presence of two pathogenic factors prompted us to attempt to distinguish the effects triggered by mutant protein from those caused by mutant RNA in cellular models of polyglutamine diseases. We used the SLIP (Synthesis of Long Iterative Polynucleotide) method to generate plasmids expressing long CAG repeats (forming a hairpin structure), CAA-interrupted CAG repeats (forming multiple unstable hairpins) or pure CAA repeats (not forming any secondary structure). We successfully modified the original SLIP protocol to generate repeats of desired length starting from constructs containing short repeat tracts. We demonstrated that the SLIP method is a time- and cost-effective approach to manipulate the lengths of expanded repeat sequences.

## 1. Introduction

At least fifteen human hereditary neurological diseases result from the expansion of unstable triplet repeats in single genes [1]. Nine of these disorders, mostly neurodegenerative, are caused by expanded CAG repeats located in translated sequences [2]. This group of disorders, known as polyglutamine (polyQ) diseases, includes Huntington’s disease, spinocerebellar ataxia types 1, 2, 3, 6, 7, and 17, spinobulbar muscular atrophy, and dentatorubral-pallidoluysian atrophy. The main type of pathogenic mechanism associated with this group of disorders is a mutant protein gain-of-function [2]. However, there is growing evidence for the contribution of mutant transcript toxicity, which resembles, to the same extent, the toxicity observed in myotonic dystrophy type 1 [3,4,5].

One way to assess the molecular mechanisms of pathogenesis in this group of disorders is to generate and investigate cellular models of polyQ diseases expressing repeated sequences of determined length and structure [6,7,8]. The existing models developed for this purpose were recently reviewed [5]. The development of such models typically requires assembly of genetic constructs from which the repeats are expressed. However, manipulating the length of CAG repeats encoding a polyQ tract in a controlled way is not simple. This is due to the hairpin structure formed by the repeats [9], DNA polymerase slippage on repeated sequences [10], and their instability in cells [11].

To address these problems, several methods of cloning repeated sequences have been developed. The commonly used method involves self-priming of two oligonucleotides, one containing CAG repeats and another containing complementary CTG repeats. The method has several modifications which differ in the PCR conditions used [12,13,14,15,16,17]. Additionally, PCR-free methods have been proposed, which involve repeated cycles of plasmid digestion with restriction enzymes and ligation of another repeat module [18]. Another approach involves the insertion of mixed CAG/CAA repeats into CAG repeat tracts using SII-type restriction enzymes to increase the repeats’ stability [19].

In this study, we assessed the *in vitro* CAG repeat expansion method known as SLIP (Synthesis of Long Iterative Polynucleotide) [15]. This method involves plasmid digestion with a restriction enzyme, a single PCR cycle, and bacterial transformation. We optimized SLIP to expand CAG, CAA and mixed (CAG)_3_CAA repeats. Both CAG and CAA triplets encode glutamine, although the codon usage of CAG is three times higher than that of CAA in human cells [20]. In the ATXN2 and TBP transcripts implicated in spinocerebellar ataxia type 2 and type 17, most of the normal variants of these genes contain CAA-interrupted CAG repeats [21,22,23,24,25,26,27,28], which have been shown to strongly alter the structures formed by pure CAG repeats. However, we could not take advantage of these natural repeat insertion patterns because of the restrictions of the SLIP method [29].

Thus, the main reason for performing our SLIP experiments with this particular set of repeated sequence motifs is the ability of their long tracts to form different structures in transcripts. The CAG repeats form long, undisturbed hairpin structures, the CAA-interrupted CAG repeats form multiple short and less stable hairpins, and long stretches of CAA repeats remain single-stranded [30]. With cellular models expressing such variants of the glutamine-coding repeats, it should be possible to distinguish between the effects caused by structure-dependent RNA toxicity and protein toxicity in polyQ diseases [5]. The importance of such an approach can be stressed by the lack of correlation between the presence of polyQ inclusions and disease, suggesting that other mechanisms, such as RNA-based toxicity, are likely to be involved.

## 2. Results and Discussion

### 2.1. Experimental Design

We intended to create genetic constructs expressing the human *ATXN3* gene, which is responsible for spinocerebellar ataxia type 3 and contains three types of repeated sequence of a defined length of approximately 60, 90, and 120 triplet repeats. The starting constructs required by the SLIP method were prepared by using standard molecular cloning techniques. The construct bearing human cDNA for *ATXN3*, containing 69 CAG repeats and flanked by *Bsm*BI and *Eco*0109I sites, was cloned into pGEM^®^-T Easy vector (Promega, Madison, WI, USA) as described previously [31].

To generate the starting constructs of pure CAA repeats and CAA-interrupted CAG repeats, we used synthetic oligonucleotides containing 20 such repeats. Oligonucleotides were annealed and cloned into a 69 CAG construct by digesting the original repeat tract with restriction enzymes and ligating the CAA or CAA-interrupted CAG repeat tract into the construct. This procedure swapped the original 69 CAG repeats with either 20 CAA or 20 CAA-interrupted CAG repeats. The resultant constructs were then subjected to repeat tract expansion by using the SLIP method. The CAG, CAA, and CAA-interrupted CAG repeat constructs were digested with either *Bsm*BI or *Eco*0109I restriction enzymes, respectively, 48 bp upstream and 3 bp downstream of the repeat tract. Pairs of single enzyme digestion products from each starting construct were then combined, subjected to one PCR cycle, and used for bacterial transformation. The colony PCR products were analyzed on agarose gels to assess repeat sequence length changes.

**Figure 1:**
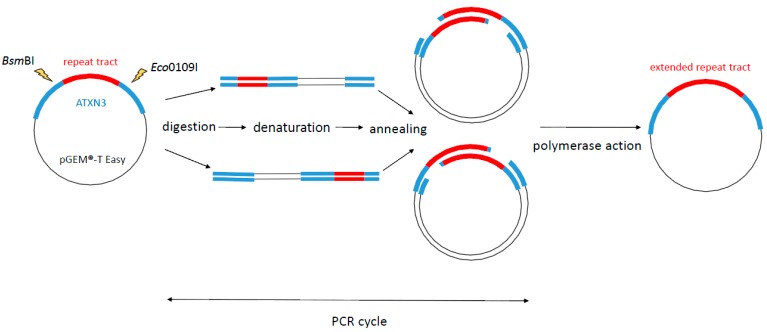
Extension of a repeat tract by using the SLIP method. Starting constructs are independently digested with enzymes that digest upstream and downstream sites of the repeat tract. The digestion products are then combined and subjected to a single PCR cycle. Repeat extension by gap-filling is carried out by using a polymerase that extends the products of different digestions which repeated sequences are not perfectly aligned. The polymerase first removes mismatched sequences immediately adjacent to the repeat tract and then extends the 3ʹ end, resulting in repeat tract elongation [15].

The SLIP method requires the presence of a repetitive sequence module to be effective. Products of independent, parallel digestions anneal to each other imperfectly, which allows the DNA polymerase to extend the repeat tract. The polymerase has to exhibit 3ʹ exonuclease activity to remove the non-annealed fragments of the specific sequence flanking the repeat tract (Figure 1).

**Figure 2:**
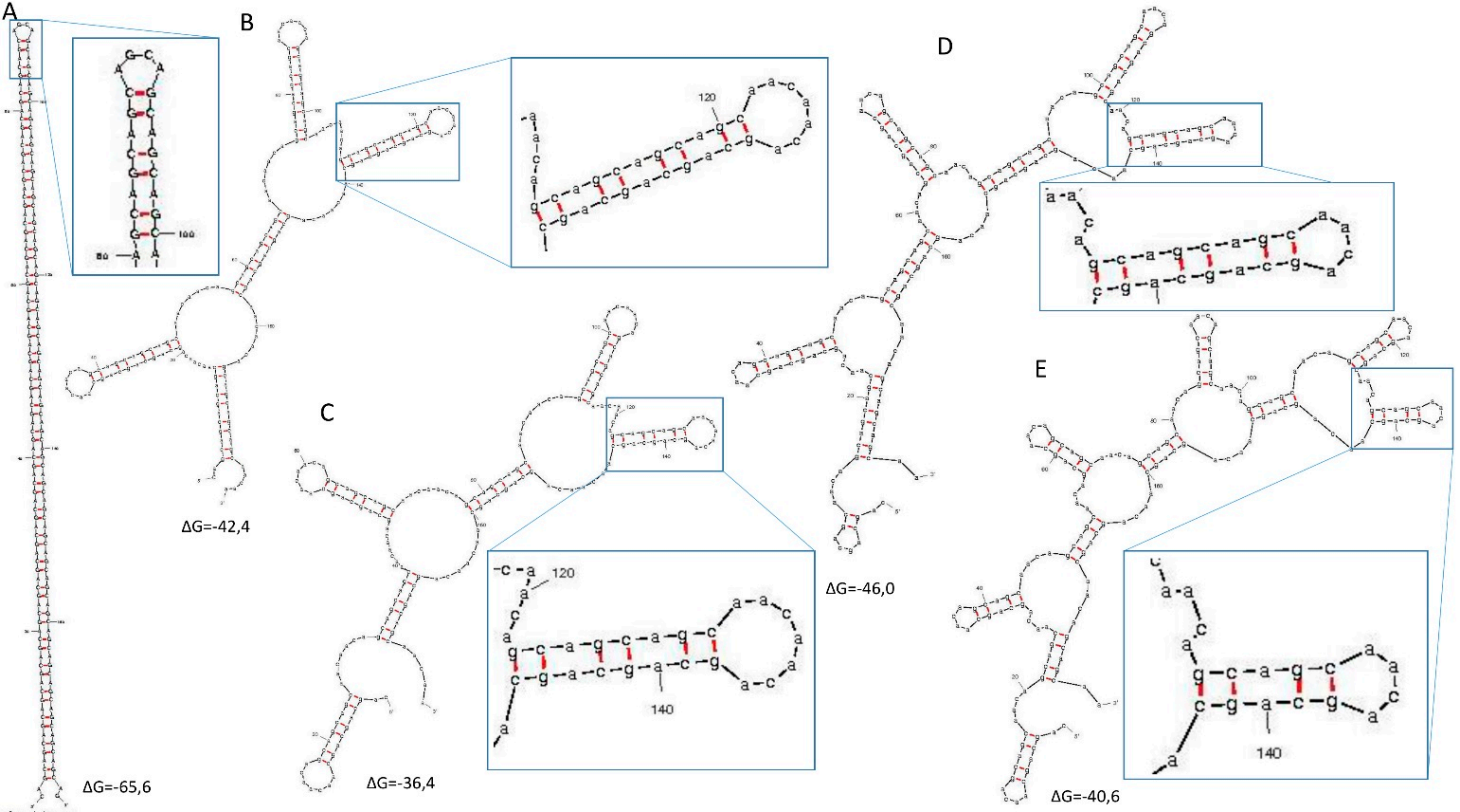
RNA structures of different repeat tracts containing 60 repeats predicted using Mfold [32,33,34]. Predictions were made using default settings, and structures having the lowest free energy of formation are presented. (**A**) Hairpin structure predicted for the pure CAG repeat tract; (**B**) the (CAG)_4_(CAA)_2_ repeat tract; (**C**) the (CAG)_3_(CAA)_2_ tract; (**D**) the (CAG)_3_CAA tract; and (**E**) the (CAG)_2_CAA tract.

The pattern of CAG repeat interruption by CAA triplets was chosen from several considered variants, including (CAG)_4_(CAA)_2_, (CAG)_3_(CAA)_2_, (CAG)_3_CAA, and (CAG)_2_CAA, based on Mfold-predicted structures of these variants in transcripts (Figure 2). For SLIP, we selected the sequence motif (CAG)_3_CAA (variant D) due to its low frequency of CAA triplets, having lower codon usage than CAG, the second smallest repeat module size, and sufficient hairpin structure destabilizing ability [29].

### 2.2. SLIP (Synthesis of Long Iterative Polynucleotide) Performance

**Figure 3:**
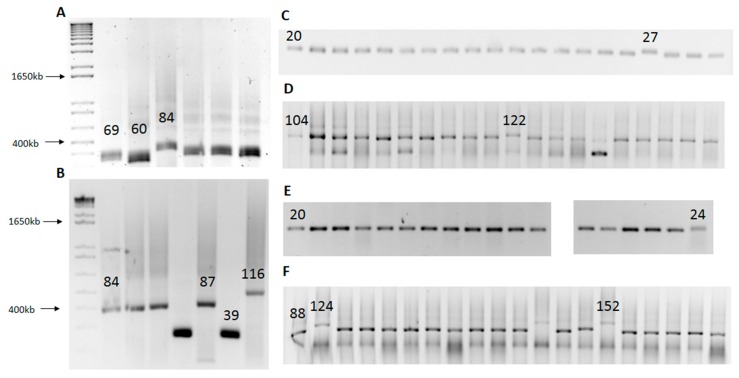
Screen of *ATXN3* constructs generated by using the SLIP method. SLIP of *ATXN3* with, (**A**) 69 CAG repeats; (**B**) 84 CAG repeats; (**C**) 20 CAA repeats; (**D**) 104 CAA repeats; (**E**) 20 CAA-interrupted CAG (1:3 ratio) repeats; and (**F**) 88 CAA-interrupted CAG (1:3 ratio) repeats. The numbers above the bands indicate the length of the repeat tract confirmed by sequencing. The first PCR product in the gel represents a control PCR product from a construct subjected to SLIP.

By performing multiple rounds of SLIP, it was possible to generate numerous variants of starting constructs that differed in repeat sequence length. Each SLIP round takes no more than three days. Day 1 involves construct digestion with restriction enzymes, PCR and bacterial transformation. Day 2 involves screening of clones by colony PCR and setting up bacterial cultures for DNA preparation. Day 3 involves DNA preparation; at this point, the system is ready for the next round of SLIP. The extent of the repeat length change is difficult to control or predict. SLIP causes not only repeat tract expansion but also contraction. When repeat sequence changes occur at a low frequency, it is necessary to screen more clones (sometimes more than 100) to find one that is expanded or contracted. Representative gels from SLIP experiments with our three types of repeats are presented in Figure 3. Panels A and B show the raw SLIP data for the CAG repeat, panels C and D show such data for the CAA repeat, and panels E and F for the (CAG)_3_CAA repeat.

Table 1 represents the outcomes of consecutive rounds of SLIP experiments performed with three types of repeats.

**Table 1:** Results of a series of SLIP experiments showing the efficiency of elongation of a particular repeat tract. L, the number of elongated clones; S, the number of shortened clones; total, the number of screened clones; “-” indicates that SLIP was not performed. When several variants of repeat length were obtained and confirmed by sequencing, the longest one was used in additional experiments. For 104 CAA and 36 CAG/CAA variants, two SLIP experiments were performed.

CAG	L	S	Total	CAG/CAA	L	S	Total	CAA	L	S	Total
69	3	3	25	20	1	0	85	20	2	0	95
84	2	2	18	24	1	1	95	25, 27	2	1	228
87, 116	-	-	-	28	2	0	114	30, 33	1	0	114
				36	4	1	114	36	1	0	114
					7	2	114				
				40, 44, 48, 52	2	3	114	53	2	2	228
				80, 88	7	2	114	58	1	2	114
				96, 108, 116, 124, 152	-	-	-	104	6	3	114
									1	5	114
								143, 150	-	-	-
								122			

As shown in this table, the expansion size is random, and short repeats are less prone to expand than long repeats, which is consistent with the nature of the SLIP method (Figure 1). Short tracts offer fewer possibilities for alternative annealing during the PCR cycle and, thus, their elongation is less likely. All CAG repeat variants were obtained using the original SLIP method [15].

We found that expansion of 20 CAA and 20 CAA-interrupted CAG repeats did not occur under conditions used to successfully elongate the 69 CAG starting construct (annealing temperature 55 °C). Therefore, we performed SLIP with two short starting constructs at lower annealing temperatures of 50, 45, and 40 °C. Only at the two latter conditions were expansion products detected. Two expanded products containing 27 and 25 repeats were obtained at 45 °C from the 20 CAA construct and one with 28 repeats from the 20 CAA-interrupted CAG construct. The results of SLIP were also compared for the 104 CAA repeat tract annealed at two different temperatures. At 40 °C, six elongated repeats and three contracted repeats were formed. The two longest expansions contained 143 and 150 CAA repeats. At 45 °C, one elongated clone containing a 122 CAA repeat tract, and five contracted clones were generated.

**Figure 4:**
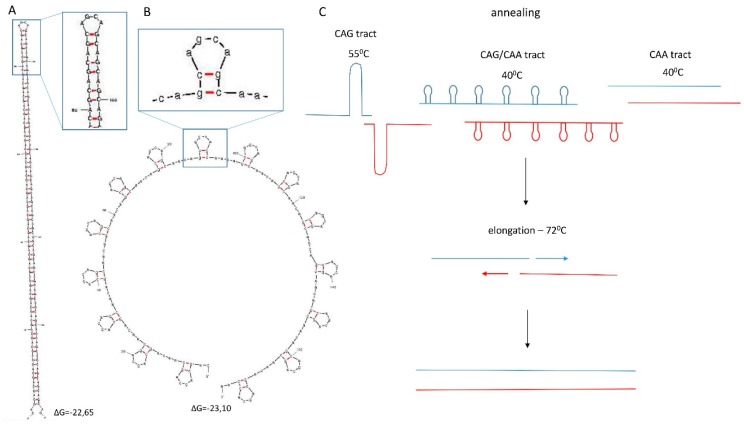
Structure of CAG and (CAG)_3_CAA repeat tracts predicted for 60 triplets using Mfold (DNA folding form), and hypothetical hairpin structure behavior during SLIP. Red and blue colors indicate products of *Bsm*BI and *Eco*0109I restriction enzymes digestions. Predictions were performed at default settings using an annealing temperature corresponding to the temperature used during the PCR cycle. (**A**) CAG repeat structure at 55 °C; (**B**) (CAG)_3_CAA repeat structure at 40 °C. Only the lowest energy structures are presented; (**C**) hypothetical behavior of repeat tracts during SLIP. At annealing temperature, hairpin structures are relatively stable, with ΔG = −22.65 kcal/mol for the CAG tract and ΔG = −23.15 kcal/mol for the CAG/CAA tract; at 72 °C, the ΔG rises to −6.1 and 0.62, respectively, making hairpins less stable and allowing polymerase to function [32,33,34].

Different types of repeats, *i.e.*, CAG, CAA, and (CAG)_3_CAA, appear to have different abilities to change their length during SLIP, which can be noted by comparing the number of expanded and contracted clones with the total number of screened clones. For example, from a 69 CAG starting tract, SLIP generated six clones with changed repeat length out of 25 clones screened. For a 58 CAA starting tract, three such clones were detected out of 114 screened, and from a 52 CAA-interrupted CAG starting tract, five out of 114 (Table 1). The tracts most susceptible to variations in length were the CAG repeat, followed by the (CAG)_3_CAA repeat and then by the pure CAA repeat. This finding may reflect that different structures formed by these three types of DNA repeats under the conditions of the experiment. Structure prediction shows that the CAG repeat tract forms a long hairpin structure (Figure 4A), (CAG)_3_CAA forms numerous shorter hairpins separated by single-stranded fragments (Figure 4B), and the CAA repeat, as in RNA, does not form any secondary structure. Hairpin structure formation probably facilitates imperfect alignments of repeated sequences, providing more room for expansion and contraction events. Figure 4C presents selected hypothetical structures of annealed restriction enzyme digestion products formed by three types of repeats used for SLIP. These models explain the varying expansion capabilities of these repeats.

## 3. Experimental Section

### 3.1. SLIP (Synthesis of Long Iterative Polynucleotide)

SLIP was performed as described previously by Takahashi *et al.* [15], with modifications described in the main text. Briefly, DNA constructs were digested in parallel using *Eco*0109I FastDigest (Thermo Scientific, Waltham, MA, USA) and *Bsm*BI FastDigest (Thermo Scientific, Waltham, MA, USA) enzymes for 40 min at 37 °C, followed by 10 min at 65 °C for enzyme inactivation. Digestion products were then combined and subjected to one PCR cycle followed by DH5α bacterial transformation.

### 3.2. Colony PCR

Colony PCR was performed using the forward primer 5ʹGGAAGAGACGAGAAGCCTAC and the reverse primer 5ʹTCACCTAGATCACTCCCAAGT. The PCR cycling conditions were an initial denaturation at 95 °C for 5 min, denaturation at 95 °C for 30 s, annealing at 60 °C for 15 s, extension at 72 °C for 30 s × 30 cycles and a final elongation at 72 °C for 7 min. The reaction mixture in the final volume of 5 µL contained 0.5 µM forward primer, 0.5 µM reverse primer, 1 mM dNTP mix, 1.25 mM Mg, 1 × GoTaq Flexi buffer and 0.0625 units GoTaq G2 Flexi polymerase (Promega, Madison, WI, USA). PCR products were than analyzed on an 1% agarose gel.

### 3.3. Preparation of Starting Constructs

Synthetic oligonucleotides with *Eco*0109I and *Bsm*BI ends were synthesized (IBB Warsaw) (Table 2). The oligomers were annealed by heating and gradual cooling. The oligomers were subsequently cloned into the 69 CAG starting constructs that were previously digested with *Eco*0109I FastDigest (Thermo Scientific, Waltham, MA, USA) and *Bsm*BI FastDigest (Thermo Scientific, Waltham, MA, USA) enzymes and purified by excising agarose gel (TAE buffer: 40 mM Trizma base (Sigma-Aldrich, St. Louis, MO, USA), 20 mM glacial acetic acid (Avantor Performance Materials Poland S.A., Gliwice, Poland), 1 mM EDTA (Avantor Performance Materials Poland S.A., Gliwice, Poland) using the Gel-Out Kit (A&A Biotechnology, Gdynia, Poland). Ligation was performed overnight at 4 °C with 50 ng of vector and a 6:1 insert/vector molar ratio using T4 DNA ligase (Promega, Madison, WI, USA). The next day, the entire reaction mixture was used to transform DH5α bacteria, and the obtained colonies were screened by colony PCR as described above. Plasmids from colonies containing constructs with an appropriate length of repeat tract were miniprepped using the Plasmid Mini Kit (A&A Biotechnology, Gdynia, Poland). The nucleotide sequence of the obtained constructs was confirmed by DNA sequencing.

**Table 2:** Sequences of synthetic oligonucleotides. Repeat tracts are shown in red and restriction sites are underlined.

Repeated Motif	Oligonucleotide Sequence
(CAG)_3_CAA	5ʹcggaagagacgagaagcctactttgaaaaacagcagcaaaagcagcaacagcagcagcaacagcagcagcaacagcagcagcaacagcagcagcaacagcagcagcaacgg (111 nt)
(CAG)_3_CAA	5ʹgtcccgttgctgctgctgttgctgctgctgttgctgctgctgttgctgctgctgttgctgctgctgttgctgcttttgctgctgtttttcaaagtaggcttctcgtctct (107 nt)
CAA	5ʹcggaagagacgagaagcctactttgaaaaacagcagcaaaagcagcaacaacaacaacaacaacaacaacaacaacaacaacaacaacaacaacaacaacaacaacaacgg (111 nt)
CAA	5ʹgtcccgttgttgttgttgttgttgttgttgttgttgttgttgttgttgttgttgttgttgttgttgttgctgcttttgctgctgtttttcaaagtaggcttctcgtctct (107 nt)

## 4. Conclusions

The SLIP method is fast and easy to perform; the entire cycle of repeat length variant synthesis and analysis can be completed within three days. We found this method to be very effective in generating ~60, ~90, and ~120 repeat length variants of CAG, CAA, and (CAG)_3_CAA repeat tracts. The latter two repeats were expanded by SLIP for the first time, starting from constructs containing very short repeat tracts of only 20 repeats. In these cases, the repeat length changes occurred less frequently, which required screening higher numbers of clones. For the SLIP method, the repeat tract needs to be flanked by nearby restriction enzyme cleavage sites. In our system, the restriction enzyme sites were located 48 bp upstream and 3 bp downstream of the repeat tract; therefore, the SLIP method can tolerate this distance. The exact outcome of the SLIP application, in terms of the size of the expansion or contraction, is difficult to control. In our study, the annealing temperature used in the PCR step was the major factor affecting the SLIP results.

The SLIP method does not need sub-cloning steps and may be used to manipulate the length of the repeat tract directly inside the gene construct. Moreover, SLIP has a higher repeat generation limit compared to PCR-based methods [15]; however, it requires starting constructs which may be considered disadvantageous. In comparison to PCR-free methods, SLIP enables generation of the desired repeat length tracts in fewer steps and is less laborious.

In conclusion, our results demonstrate that SLIP is indeed a versatile method for extension and/or contraction of repeated sequences, and that this method is not restricted to manipulation of long repeats forming stable secondary structures. The ATXN3 constructs, described in this study, have been sub-cloned into expression vectors and transfected human neuroblastoma SK-N-MC cells have been analyzed for expression of RNA toxicity markers (manuscript in preparation).
